# Tumor-Associated Macrophages and Their Functional Transformation in the Hypoxic Tumor Microenvironment

**DOI:** 10.3389/fimmu.2021.741305

**Published:** 2021-09-16

**Authors:** Zicong He, Shuixing Zhang

**Affiliations:** Department of Radiology, First Affiliated Hospital of Jinan University, Guangzhou, China

**Keywords:** tumor hypoxia, hypoxic tumor microenvironment, tumor-associated macrophages, macrophage polarization, macrophage functional transformation

## Abstract

Tumor-associated macrophages (TAMs) are some of the most abundant immune cells within tumors and perform a broad repertoire of functions *via* diverse phenotypes. On the basis of their functional differences in tumor growth, TAMs are usually categorized into two subsets of M1 and M2. It is well established that the tumor microenvironment (TME) is characterized by hypoxia along with tumor progression. TAMs adopt an M1-like pro-inflammatory phenotype at the early phases of oncogenesis and mediate immune response that inhibits tumor growth. As tumors progress, anabatic hypoxia of the TME gradually induces the M2-like functional transformation of TAMs by means of direct effects, metabolic influence, lactic acidosis, angiogenesis, remodeled stroma, and then urges them to participate in immunosuppression, angiogenesis and other tumor-supporting procedure. Therefore, thorough comprehension of internal mechanism of this TAM functional transformation in the hypoxic TME is of the essence, and might provide some novel insights in hypoxic tumor immunotherapeutic strategies.

## Introduction

The tumor microenvironment (TME) is now recognized as a major contributor to cancer progression. Hypoxia, resulting from an imbalance between oxygen supply and consumption (1), is an intrinsic property of the TME. The rapid proliferation of cells in the tumor mass necessitates extensive vascularization to sustain an adequate oxygen supply; however, tumor vessels are usually immature, disorganized, and hyperpermeable (2), leading to intratumoral oxygen deprivation. Cancer cells adapt to the resultant hypoxic microenvironment mainly *via* the hypoxia-inducible factor (HIF) signaling pathway, which regulates the expression of genes that contribute to immune evasion and malignant progression (3, 4). However, such inhospitable conditions are not favorable for infiltrating immune cells and promote their immunosuppressive functions (5).

Macrophages, which originate from circulating bone marrow-derived monocytic precursors, are among the most abundant immune cells within tumors and can be polarized into different phenotypes, each of which is associated with different and diverse functions (6, 7). According to their functional differences, these tumor-associated macrophages (TAMs) can be broadly categorized into two subsets, namely, M1 (pro-inflammatory and anti-tumor) and M2 (anti-inflammatory and pro-tumor) (8). M1-like TAMs are activated by IFN-γ, lipopolysaccharide, IL-1β, TNF, and/or GM-CSF and can recognize and destroy malignant cells *via* phagocytosis and cytotoxicity, in addition to producing pro-inflammatory cytokines that stimulate anti-tumor immunity (9–11). In contrast, M2-like TAMs are induced by Th2 cytokines such as IL-4, IL-10, IL-13, and/or M-CSF, and can favor tumor growth and promote TME remodeling by producing growth factors, immunosuppressive factors, pro-angiogenic molecules, and proteases (9, 12–14). However, this simplified distinction of M1/M2 polarization cannot strictly delineate the phenotypic and functional boundaries of TAMs as these cells are both highly dynamic and heterogeneous within and across tumors (15). TAMs have an extraordinary degree of plasticity, which enables them to finely modulate themselves in response to microenvironmental changes and thereby orchestrate various aspects of the TME (7, 15). Hypoxia is a microenvironmental cue that induces the tumor-supporting transformation of TAMs, an effect that is associated with disease progression and resistance to therapy (16). This highlights the need to integrate TAM-related hypoxic stress into tumor immunotherapy.

**Figure 1:**
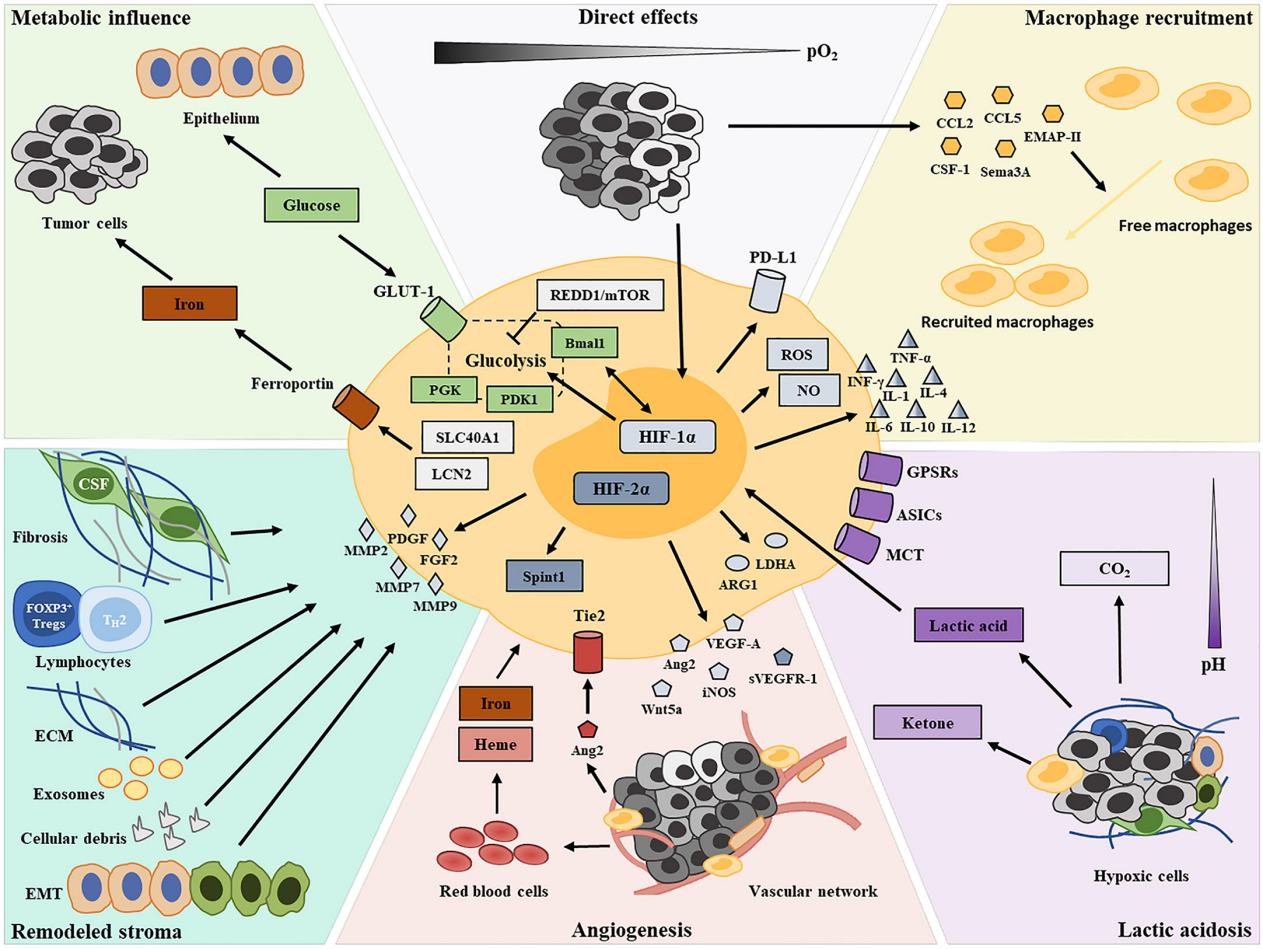
Graphical summary depicting the contributors of TAM functional transformation in the hypoxic TME.

Here, we review the known mechanistic effects of a hypoxic TME on TAM functional transformation (**Figure 1**) and provide insights into immunotherapeutic strategies targeting hypoxic macrophages.

## Pro-Tumor Transformation of TAMs in the Hypoxic TME

### Hypoxia-Driven TAM Recruitment

Due to unbalanced growth and a disorganized microvasculature, there is significant heterogeneity in oxygen content in a tumor mass. The hypoxic condition induces the production of a broad array of migratory stimulating factors, such as VEGF, CCL2, CCL5, CSF-1, EMAP-II, endothelin-2, SEMA3A, oncostatin M, and eotaxin, in tumor cells and the stroma within oxygen-deprived regions (17–24), resulting in macrophage recruitment and entrapment (25). When macrophages are recruited in hypoxic tumor areas, their polarization can be altered to an M2-like pro-tumor phenotype *via* the activity of the above-mentioned hypoxic tumor cell-derived cytokines (20, 24). A recent study revealed that neuropilin-1 (NRP-1) expression is significantly upregulated in hypoxic areas and induces pro-tumor phenotypes in recruited macrophages (26). Consequently, there is a greater abundance of M2-like TAMs at the invasive margin of tumors, where the hypoxic status is more severe, compared with that at the tumor center (27).

### Direct Effects of Hypoxia

Hypoxia may also direct TAM polarization by affecting gene expression profiles. HIFs are key hypoxia-responsive transcription factors, the expression of which is upregulated in macrophages (28). Two isoforms of HIF—HIF-1 and HIF-2—elicit overlapping but sometimes opposing effects on macrophage transcriptional profiles, which endow macrophages with plasticity and shape their versatile phenotypes (29, 30). HIF activity in macrophages is dependent on the type of cytokine stimulus (31), with HIF-1α reported to be activated by Th1 cytokines and HIF-2α by Th2 cytokines. Additionally, HIF-1α and HIF-2α, *via* the regulation of respectively the inducible nitric oxide (NO) synthase and the arginase 1 genes, coordinately regulate NO availability to guide macrophage functional phenotypes (31). HIF-1α and HIF-2α are known to participate in the inflammatory function of macrophages. Macrophages sense changes in oxygen concentrations and then mediate IFN-γ production *via* HIF-1α, thereby enhancing their phagocytic functions and antigen presentation abilities (32). Meanwhile, HIF-1α promotes the production of inflammatory molecules in a TLR4-dependent fashion, including granule proteases, antimicrobial peptides, TNF-α, IL-1, IL-4, IL-6, and IL-12, thereby regulating the killing capacity of macrophages (33, 34). *In vitro* findings indicated that the absence of HIF-1α in macrophages leads to reduced ARG1 expression and the consequent suppression of T-cell activation (35). Additionally, there is evidence to indicate that HIF-1α affects the inflammatory function of macrophages by regulating their glycolytic capacity under hypoxic conditions (36). The contributions of HIF-2α to pro-inflammatory cytokine expression in hypoxic macrophages have also been documented (37). However, unlike HIF-1α, the regulation of inflammation by HIF-2α involves neither the production of NO nor the expression of costimulatory molecules (33, 37). Furthermore, HIF-1α and HIF-2α were found to exert antagonistic functions in angiogenesis. The role of HIF-1α as a positive regulator of macrophage-derived VEGF is well established (38). The knockout of HIF-1α in TAMs can attenuate their pro-angiogenic responses (39). In contrast, HIF-2α upregulates the production of soluble VEGF receptor 1 (sVERFR-1) by macrophages (40, 41). sVERFR-1 is an alternatively spliced variant of the membrane-bound VEGFR-1 expressed on endothelial cells and acts as a negative regulator of VEGF in tumor angiogenesis (40). Furthermore, HIF-1α was recently reported to upregulate the expression of PD-L1 in tumor-infiltrating macrophages, thereby promoting the establishment of an immunosuppressive TME (42). A recent study found that macrophage-derived HIF-2α regulates the expression of the serine protease inhibitor Kunitz type 1 (*SPINT1*), which contributes to the tumor-suppressive functions of TAMs in breast cancer development (43). Nonetheless, the latest evidence from single-cell RNA sequencing revealed that macrophages within both tumors and normal tissues do not show defined M1 or M2 polarization signature gene expression (44). The multifarious functional phenotypes of TAMs in hypoxic tumors might not be entirely dependent on gene expression profiles, but may also be influenced by the local environment.

At the early stages of oncogenesis, infiltrating macrophages adopt an M1-like phenotype that promotes the destruction of tumor cells and the inhibition of angiogenesis, concomitant with the activation of the inflammatory response (45). However, chronic inflammation resulting from M1-like TAM activity can accelerate genomic instability in malignant cells and serve as a driver of tumor progression (46, 47). As tumors progress, increasing levels of hypoxia lead to reduced secretion of pro-inflammatory mediators (e.g., IL-1β, TNF-α, and CCL17) by M1-polarized macrophages and facilitates macrophage differentiation toward the M2-like phenotype (48). Although hypoxia does not directly alter the relative abundance of macrophage subsets, it induces a pro-tumor gene expression profile in the M2-like macrophage subset (49), including the expression of growth factors (e.g., FGF2, PDGF, and VEGF) (50, 51), angiogenic molecules (e.g., VEGF, FGF2, CXCL8, and IL-8) (52), angiogenic modulators (e.g., COX2 and iNOS) (52), and matrix metalloproteinases (e.g., MMP2, MMP7, and MMP9) (53, 54). Furthermore, hypoxia can reportedly promote an increase in CCL20 expression in TAMs through the ERK/NF-κβ pathway, leading to the accumulation of CCR6^+^ Foxp3^+^ T regulatory cells (Tregs) (55). Although TAMs show no differences in M1 and M2 polarization capacity, they tend to exert M2-like pro-tumor functions in the hypoxic TME (35).

### Metabolic Influence of Hypoxia

Hypoxia is known as a metabolic cue that shapes macrophage functional phenotypes within the TME. M1-like macrophages usually employ glycolytic metabolism for their energy supply and have a robust capacity for reactive oxygen species (ROS) production; in contrast, M2-like macrophages generally utilize oxidative phosphorylation to fuel their longer-term tissue repair functions (56). The crucial role of HIF-1α in regulating the glycolytic capacity of macrophages, as well as their survival and function, in the hypoxic TME has been documented (36). The expression of the glycolytic enzyme phosphoglycerate kinase (PGK) and glucose transporter 1 (GLUT-1) is markedly reduced in macrophages with deletion of myeloid HIF-1α, as is the cellular ATP pool, which leads to an impaired inflammatory response (33, 36). There is some evidence to suggest that pro-inflammatory macrophages redirect pyruvate away from pyruvate dehydrogenase (PDH) in a NO-dependent and HIF-1α-independent manner, thereby promoting their metabolic reprogramming (57). Pyruvate dehydrogenase kinase, isozyme 1 (PDK1), induced by HIF-1α in mildly hypoxic condition, has been found to regulate glycolytic reprograming of macrophages through the redirection of pyruvate flux into lactate, while leaving cytochrome c oxidase activity unaffected (58). Such active glycolysis promotes the redistribution of intracellular ATP, and plays an essential role in macrophage migratory capacity (58). However, long-term hypoxia in tumors still exerts a negative influence on TAM metabolism. Mammalian target of rapamycin (mTOR) functions as an integrative rheostat that couples cellular activation to nutrient sensing and metabolic status (59, 60). Hypoxia drives the upregulation of regulated in development and DNA damage response 1 (REDD1), an inhibitor of mTOR, which strongly hinders glycolysis in TAMs and reduces their metabolic competition with endothelial cells (61, 62). Such a REDD1/mTOR metabolic shift in TAMs culminates in endothelial cell hyperactivation, with the consequent formation of an abnormal vascular network (61, 62). A significant reduction in microRNA-30c levels is also observed in hypoxic TAMs, which impairs both mTOR activity and glycolysis, thereby inhibiting TAM M1-like polarization (63). BMAL1 is known as a molecular clock that regulates mitochondrial metabolism under metabolic stress in macrophages. A recent study found that BMAL1/HIF-1α crosstalk regulates macrophage energy metabolism, while metabolic dysregulation due to aberrant HIF-1α activation in TAMs contributes to an immunosuppressive TME (64).

Iron is an essential nutrient for malignant cell growth and proliferation and also contributes to both tumor progression and metastasis (65). Most iron is recycled and released to tissues by macrophages *via* erythrophagocytosis (66). M2-like TAMs exhibit a gene expression profile associated with iron efflux (increased ferroportin levels and reduced ferritin levels), whereas M1-like TAMs favor iron retention (67, 68). Tumor hypoxia supports such an iron-donor phenotype by upregulating solute carrier family 40, member 1 (SLC40A1) and lipocalin 2 (LCN2) expression in TAMs, resulting in increased iron availability in the TME and improved iron uptake by malignant cells (69–71).

### Lactic Acidosis After Hypoxia

It is well established that the hypoxic TME is characterized by acidosis. Hypoxic tumor cells mainly obtain energy *via* anaerobic glycolysis, leading to increased concentrations of lactic acid (72). Meanwhile, such fermentative metabolism occurs in highly proliferating cells even in the presence of oxygen, known as the “Warburg Effect” (72). This byproduct of aerobic or anaerobic glycolysis (together with M-CSF) downregulates the NF-κB pathway, reduces the secretion of both NO and inflammation-related cytokines (such as TNF-α and IL-1), while simultaneously inducing the expression of *VEGFA*, *ARG1*, and other M2-associated genes (73–75). Besides, M2−like TAMs altered by lactic acid were found to promote T−cell apoptosis through the PD−L1/PD−1 pathway (76). Recent findings have shown that a pH of 6.1 without stimulation or a pH of 6.8 with IL-4 stimulation could promote the expression of *ARG1* and *VEGFA* by macrophages *in vitro* (77, 78). These effects of tumor-cell-derived lactic acid are mediated by HIF-1α and promote TAM polarization toward an M2-like phenotype (73–75). A different study reported that lactic acid could inhibit ATP6V0d2 expression in TAMs, thereby promoting their HIF-2α-mediated pro-tumor functions (79). This suggests that lactic acid promotes the tumor-supporting phenotype of TAMs *via* the activation of HIF-1α3 and HIF-2α, albeit through distinct mechanisms. Under normoxic conditions, lactic acid normally exerts only a weak effect on TAMs. Under hypoxia, however, lactic acid greatly facilitates M2-like polarization *via* the HIF-1, Hedgehog, and mTOR pathways (80). Furthermore, G protein-coupled receptors (GPCRs) have been reported to function as key sensors of the acidification of the TME, inducing the expression of inducible cyclic AMP early repressor (ICER; transcriptional repressor), which enhances the pro-tumor transition of TAMs *via* NF-κB signaling inhibition (77, 81). Moreover, the activation of acid-sensing ion channels (ASICs) was identified as an important mediator of the endocytic functions of macrophages as well as their maturation (82). Recently, lactic acid was shown to be capable of skewing the macrophage phenotype toward the M2-like state *via* monocarboxylate channel transporter (MCT)/HIF-1α signaling (83). Lactate-derived histone lysine lactylation, a recently identified epigenetic modification, was demonstrated to induce the expression of M2-associated genes, including *ARG1* (84). Moreover, the most recent evidence has indicated that tumor-released succinate can activate succinate receptor 1 (SUCNR1) signaling to polarize TAMs toward tumor-supporting phenotypes through a SUCNR1-activated PI3K/HIF-1α axis (85).

### Angiogenesis in Hypoxic Areas

Hypoxia in the TME induces angiogenesis to meet the oxygen and nutrient needs of proliferating tumor cells. TAMs accumulate and transition into proangiogenic phenotypes in perivascular areas (86), especially those that are poorly vascularized (87). TIE2, an angiopoietin (ANG) receptor expressed by TAMs, is upregulated under hypoxic conditions and, together with ANG-2, enhances the pro-tumor functions of TAMs (88, 89). Compared with TIE2^−^ TAMs, TIE2^+^ TAMs within the same tumor express higher levels of pro-angiogenic genes, including *MMP9*, *VEGFA*, *COX2*, *WNT5A*, and *PDGFB* (90, 91). ANG-2 expression is known to be increased in hypoxic regions and serves as a chemoattractant for macrophages (89). ANG-2, secreted from tumor and vasculature cells, can enhance IL-10 and mannose receptor expression, while decreasing that of TNF-α and IL-12, thereby weakening TAM anti-tumor activity under hypoxic conditions (88, 89).

The secretion of macrophage-derived VEGF-A is also markedly increased by HIF-1α at hypoxic sites, thereby enhancing tumor angiogenesis (92, 93). In contrast, under the regulation of HIF-2α, hypoxic TAMs generate high levels of sVEGFR-1, which selectively neutralizes VEGF activity and diminishes tumor angiogenesis (40, 41). This antagonistic effect of HIF-1α and HIF-2α on angiogenesis was suggested to facilitate the redistribution of the vascular network in hypoxic tumors to meet their growth and metabolic requirements. Of note, HIF-2α is also highly expressed in normoxic macrophages, leading to enhanced transcription of proangiogenic genes (52).

Neoangiogenesis can provide oxygen and nutrients to hypoxic areas, but can also result in erythrocyte extravasation and hemolysis. The release of heme and iron from hemolytic red blood cells can help convert M2-like TAMs into pro-inflammatory M1-like TAMs that display tumor-killing activity (94).

### Hypoxia-Remodeled Stromal Components

Stromal fibrosis is a commonly occurring event in the hypoxic TME. Cancer-associated fibroblasts (CAFs) are considered to be the dominant component of fibrotic stroma and can be activated by tumor hypoxia through several mechanisms (95). These activated fibroblasts have been found to overexpress numerous pro-inflammatory cytokines (e.g., CCL2, CCL5, IL-4, IL-6, IL-8, GM-CSF, CXCL8, and CXCL14) that regulate TAM recruitment, differentiation, and activation (96). CAF-derived CXCL14 has been demonstrated to affect macrophage recruitment in tumors *via* NOS1-derived NO signaling. CAFs have also been reported to impair the maturation and differentiation of recruited macrophages, locking them in a suppressive state, through the induction of STAT3 phosphorylation (97, 98). *In vitro* observations have indicated that CAF might drive myeloid cells toward immunosuppressive differentiation *via* the production of IL-4, IL-6, and IL-8 (99).

Extensive lymphocyte subpopulations also constitute a major fraction of tumor stroma. These lymphocytes in the hypoxic TME engage the tumor-supporting activities of TAMs *via* a large array of cytokines. For instance, Th2 lymphocyte-derived IL-4 and IL-13 can enhance epidermal growth factor expression in TAMs, which promotes tumor cell metastasis, as well as the suppressive activity of TAMs, which blunts CD8^+^ T-cell responses to therapy (100, 101). Moreover, there is evidence showing that hypoxia can upregulate the expression of forkhead box P3 (FOXP3), a transcriptional activator of Tregs, through an HIF-1α-dependent mechanism (102), while FOXP3^+^ Tregs drive TAMs toward an immunosuppressive phenotype (103, 104).

Extracellular matrix (ECM), which serves as a structural scaffold for immune cell infiltration in the TME, is extensively remodeled under tumor hypoxia (105). Hyaluronic acid (HA), a primary ECM component, is associated with macrophage trafficking and tumor neovascularization (106). Hypoxia enhances the endogenous production of HA by tumor cells (107). Pro-angiogenic M2-like TAMs preferentially traffic to HA-rich areas in the TME (106). Tumor-derived HA has also been identified to trigger the transient, early activation of monocytes, thereby promoting M2-like immunosuppressive phenotypes among TAMs (108). Another study reported that periostin and collagen, both fibrosis-associated ECM components, respectively facilitated TAM recruitment *via* integrin binding (109) and promoted their M2-like polarization (110).

Cellular debris resulting from cell death is prevalent within hypoxic regions of tumors. The release of high mobility group protein B1 (HMGB1) was demonstrated to drive IL-10 production in TAMs selectively through the receptor for advanced glycation end products (RAGE), leading to an IL-10-rich milieu within the tumor (111). The recognition of apoptotic cells is also thought to suppress macrophage activation potential (112). TAMs can recognize dying tumor cells through the MER tyrosine-protein kinase (MERTK) receptor and upregulate the expression of wound-healing factors such as TGF-β, IL-10, and ARG1 that suppress anti-tumor immunity (113).

Research attention has increasingly focused on exosomes released by hypoxic tumor cells. Hypoxia can stimulate tumor cells to produce higher numbers of exosomes (114). Exosomes in hypoxic tumor areas contain large amounts of chemokines and immunomodulatory proteins, including CSF-1, CCL2, FTH, FTL, and TGF-β, which promote the differentiation of infiltrating myeloid cells toward an M2-like macrophage lineage (115). Exosomal miR-301a-3p derived from hypoxic pancreatic cancer cells was reported to promote M2-like macrophage polarization by activating the PTEN/PI3Kγ pathway (116). MiR-7a, another exosomal miRNA derived from hypoxic tumor cells, was shown to suppress several target genes of the insulin pathway, such as *INS-1* and *IGF1R*, and thus trigger M2-like TAM polarization (117), similar to that seen for miR940 from exosomes derived from ovarian epithelial carcinoma cells (118). Recently, exosomal lncRNA BCRT1 was demonstrated to promote M2-like phenotype polarization and enhance macrophage-induced tumor progression (119). Additionally, miR-1246 in hypoxic glioma-derived exosomes was shown to mediate H-GDE-induced M2-like macrophage polarization by targeting TERF2IP *via* activating and inhibiting the STAT3 and NF-κB signaling pathways, respectively (120). Hypoxic stress was also demonstrated to suppress miR101 expression, which resulted in an increase in TAM-derived IL-1α and IL-6, which, in turn, promoted lung tumor cell growth (121).

Epithelial to mesenchymal transition (EMT) is also a common phenomenon associated with stroma remodeling in hypoxic tumors, helping to foster an immunosuppressive TME and facilitating tumor progression and metastasis (122, 123). A significant correlation has been confirmed to exist between EMT and TAM infiltration in hypoxic tumor tissues (124). Zinc finger E-box binding homeobox 1 (ZEB1) plays a critical role in the EMT program by restraining epithelial differentiation *via* the inhibition of members of the microRNA-200 family (125). The high expression of ZEB1 in hypoxic regions has a positive relationship with M2-like TAM abundance, i.e., it recruits M2-like TAMs by activating CCL8 transcription (126). Moreover, high HIF-1α expression under hypoxic conditions leads to increased secretion of the cytokine IL-1β by M2 TAMs, which, in turn, enhances EMT progression (127).

## Immunotherapeutic Strategies Targeting Hypoxic TAMs

Substantial evidence supports that the hypoxia-induced immunosuppressive TME elicits a more aggressive tumor phenotype and promotes resistance to treatment (128). Several studies have reported that TAM polarization might counterproductively be skewed towards an M2-like pro-tumor phenotype after chemotherapy and radiotherapy, which contributes to tumor revascularization and relapse, while increasing levels of hypoxia after therapy could further enhance the tumor-supporting functions of TAMs (129, 130). This highlights the potential of TAMs as immunotherapeutic targets for hypoxic tumors. Macrophage-centered therapeutic strategies for treating hypoxic tumors should focus on improving the hypoxic status of the TME, inhibiting the tumor-promoting functions of M2-like TAMs, or reactivating the anti-tumor activity of M1-like TAMs.

### Improving the Hypoxic Status of the TME

As described above, the hypoxic TME is responsible for the pro-tumor transformation of TAMs. Redressing hypoxia in the TME may be beneficial for reversing the malignant TAM phenotypes and improving responses to immunotherapy. Oxygen delivery to hypoxic areas *via* nanomaterials may be an attractive means for achieving this. Various strategies for delivering O_2_ to the hypoxic TME have been reported, such as using certain oxygen carriers for transporting O_2_ to tumor sites or generating O_2_ from endogenous hydrogen peroxide *in situ* using nanocatalysts (131–134). Recently, a TAM-targeted biomimetic nano red blood cell system was designed for precise O_2_ delivery and M2-like TAM depletion within the TME (135). This nanosystem alleviated tumor hypoxia and markedly enhanced chemoimmunotherapeutic effects. Normalization of the tumor vasculature represents another possible approach for directly alleviating tumor hypoxia. Vessel normalization is now thought to be beneficial for tumor immune reprogramming (136). As is generally acknowledged, a wide spectrum of highly expressed pro-angiogenic proteins are responsible for the abnormal vasculature networks found in hypoxic tumors. Scheduling a proper dose of anti-angiogenic drugs that block these pro-angiogenic proteins or their receptors, such as VEGF/VEGFR, could help restore functional vessels, thus alleviating tumor hypoxia (137). Low-dose anti-VEGFR2 therapy has been reported to improve the perfusion of hypoxic tumors and promote an immunosuppressive-to-immunostimulatory TAM phenotype conversion (138). Counterintuitively, monotherapy with anti-angiogenic drugs at high doses might be counterproductive owing to the associated excessive pruning of tumor vessels (137). Modification of the HIF signaling pathway might be another way of alleviating hypoxia in the TME. Vorinostat (suberoylanilide hydroxamic acid, SAHA) is a histone deacetylase inhibitor that has been approved by the United States Food and Drug Administration (FDA) and has been demonstrated to negatively regulate the expression and function of HIF-1α through the inhibition of an eIF3G-dependent translation mechanism (139). Meanwhile, topotecan, a FDA-approved topoisomerase I inhibitor, has been shown to inhibit HIF-1α protein accumulation through a DNA damage-independent mechanism and thus delay both angiogenesis and tumor growth (140).

### Inhibiting the Tumor-Promoting Functions of M2-like TAMs

The depletion of M2-like TAMs represents a possible therapeutic approach for lessening pro-tumor functions. Liposomal clodronate treatment was shown to attenuate lung cancer progression through depleting TAMs (141). Additionally, trabectedin (ET-743), originally developed as an anti-proliferative agent for soft tissue sarcoma and relapsed ovarian cancer, was reported to activate the extrinsic apoptotic pathway *via* TRAIL receptors, followed by TAM depletion in tumors (142). However, anti-cancer therapy with trabectedin might elicit undesirable effects on monocyte/macrophage-mediated host defenses because of the indiscriminate depletion of macrophages (142). As a consequence, molecular-targeting has emerged as a promising direction for M2-like TAM depletion. Cieslewicz and colleagues constructed an M2-targeting fusion peptide to selectively exhaust M2-like TAMs, thereby reducing systemic damage (143).

Because macrophages are recruited and entrapped in hypoxic areas of tumors by tumor- and stroma-derived chemoattractants, preventing macrophage recruitment *via* pharmacological modulation may be another effective treatment method for inhibiting the pro-tumor functions of TAMs. Several antibodies selectively targeting chemoattractant receptors, including CCL2R, VEGFR2, and CSF-1R, have been shown to reduce macrophage infiltration and suppress tumor growth (144–146). Accordingly, interfering pharmacologically with other macrophage chemoattractants, such as CXCL12 and CCL5, as a means of inhibiting tumor growth merits further investigation (147, 148).

### Reactivating the Anti-Tumor Activity of M1-like TAMs

As mentioned above, M1-like TAMs possess anti-tumor activity, such as the ability to inhibit tumor angiogenesis as well as the activation of inflammatory responses. This suggests that repolarizing TAMs to an M1-like phenotype may be an additional supplement to the arsenal of anti-cancer therapies. One study found that zoledronic acid, a nitrogen-containing bisphosphonate used for the treatment of cancer patients with bone metastases, could convert the TAM phenotype from M2-like to M1-like by targeting the mevalonate pathway (149). Additionally, M2-like TAMs activated using CD40 agonists can reportedly reacquire antigen-presenting capabilities and become tumoricidal, resulting in the reestablishment of tumor immune surveillance and the short-term reduction of tumor volume (150). Meanwhile, it has been shown that Toll-like receptor 3 (TLR3) signaling can transform tumor-supporting TAMs into tumor suppressors by rapidly inducing the production of pro-inflammatory cytokines (151). Furthermore, there is evidence to support that the structural and functional restoration of the tumor vasculature might restore the anti-tumor functions of TAMs. It has been demonstrated that histidine-rich glycoprotein (HRG) can downregulate placental growth factor (PlGF) levels, leading to the restoration of tumor vessel functionality and TAM repolarization (152). CSF-1R inhibition has also been reported to alter TAM polarization in combination with glioma-secreted factors, including GM-CSF and IFN-γ (20). Anti-CD47-elicited antibody-dependent cellular phagocytosis might also lead to the skewing of TAM polarization toward an M1-like phenotype (153). Recent studies have found that PI3Kγ signaling represents a crucial mediator of the switching between immunostimulatory and immunosuppressive macrophage phenotypes. The selective inactivation of PI3Kγ can stimulate and prolong NF-κB activation while inhibiting that of C/EBPβ, thereby restoring the pro-inflammatory functions of macrophages (154). However, whether the anti-tumor functions of repolarized TAMs will be overridden by the hypoxic TME remains unclear and warrants further investigation.

## Concluding Remarks

Hypoxia is a critical modulator of tumor immunity. TAMs, an important component of tumor immunity, are recruited into the hypoxic regions of tumors, where they acquire a pro-tumor phenotype following direct or indirect stimulation by the hypoxic TME. TAMs subsequently become important contributors to tumor immune escape, angiogenesis, matrix remodeling, metabolic changes, and treatment resistance through a vast array of pathophysiological processes. Although hypoxia-modified gene expression profiles endow TAMs with plasticity and versatility, the interaction with the hypoxic TME finally defines their specific functions. Consequently, a close characterization of the cross-talk between the TAM functional state and other components of the TME might offer significant insight into the development of new treatment regimens. Alleviating hypoxia in the TME and the phenotypic conversion of TAMs might be the focus of future efforts for cancer immunotherapy.

## Author Contributions

Conceptualization, ZH. Investigation and Resources, ZH. Writing - Original Draft Preparation, ZH. Writing - Review and Editing, SZ. Visualization, ZH. Graphics, ZH. Supervision, SZ. and Project Administration, SZ. All authors contributed to the article and approved the submitted version.

## Funding

This research was supported by the National Natural Science Foundation of China (81871323, 81801665, 81901709) and the Natural Science Foundation of Guangdong Province (2018B030311024, 2019A1515011918).

## Conflict of Interest

The authors declare that the research was conducted in the absence of any commercial or financial relationships that could be construed as a potential conflict of interest.

## Publisher’s Note

All claims expressed in this article are solely those of the authors and do not necessarily represent those of their affiliated organizations, or those of the publisher, the editors and the reviewers. Any product that may be evaluated in this article, or claim that may be made by its manufacturer, is not guaranteed or endorsed by the publisher.
